# Electroacupuncture pretreatment alleviates myocardial ischemia/reperfusion‐induced lung injury by inhibiting the expression of HMGB1/RAGE in male rats

**DOI:** 10.14814/phy2.71066

**Published:** 2026-09-25

**Authors:** Chenlong Xie, Jiaojiao Zhang, Yujiao Zhang, Hao Chi, Yue Yong, Jiangang Song

**Affiliations:** ^1^ Department of Anesthesiology Shuguang Hospital, Shanghai University of Traditional Chinese Medicine Shanghai China; ^2^ Fuyang Institute of Technology Fuyang China; ^3^ Department of Cardiothoracic Surgery Shuguang Hospital, Shanghai University of Traditional Chinese Medicine Shanghai China

**Keywords:** electroacupuncture, inflammation, lung injury, myocardial ischemia/reperfusion, traditional medicine

## Abstract

Myocardial ischemia–reperfusion (MI/R)‐induced lung injury is a major pulmonary complication after cardiac surgery. We aimed to verify this phenomenon in rats and to investigate whether electroacupuncture (EA) pretreatment at bilateral Feishu (BL13) and Zusanli (ST36) acupoints (once daily for 3 days) exerts protective effects. In an MI/R‐induced lung injury model in SD rats, EA pretreatment significantly improved lung function and reduced pathological damage (*p* < 0.05 and *p* < 0.01, respectively). It also attenuated the pulmonary inflammatory response and decreased the expression of high mobility group box 1 (HMGB1) and receptor for advanced glycation end products (RAGE) (*p* < 0.05 or *p* < 0.01). Intratracheal administration of recombinant HMGB1 (rHMGB1) to upregulate lung HMGB1/RAGE signaling impaired the pulmonary protective effect of EA (*p* < 0.05, *p* < 0.01). Together, these findings demonstrate that EA pretreatment mitigates MI/R‐induced lung injury by inhibiting HMGB1/RAGE signaling.

## 1 INTRODUCTION

Major pulmonary complications (MPCs) pose significant challenges during cardiac surgery (Miskovic & Lumb, 2017). Intraoperative myocardial ischemia–reperfusion not only damages heart tissue but also affects distant organs, particularly the lungs, which are susceptible and show clinical symptoms first (Lai et al., 2017; Zhan et al., 2018). The occurrence of MPCs following cardiac surgery ranges from 19% to 40%, compared with non‐cardiac surgery (5%–8%) (Tafelmeier et al., 2021). Postoperative MPCs still lead to extended hospital stays and substantial financial costs despite improvements in perioperative care, both of which hinder the achievement of enhanced recovery after surgery (ERAS) goals (Mathis et al., 2019). Investigating the pathological mechanism of myocardial ischemia–reperfusion (MI/R)‐induced lung injury and exploring improved treatment methods is crucial.

The pathological mechanism of MI/R‐induced lung injury is closely linked to pulmonary inflammatory responses (Liu et al., 2022). Subsequent studies have shown that tumor necrosis factor (TNF), interleukin (IL) ‐6, and IL‐1β enhance the expression of cell adhesion molecules, leading to increased lung endothelial cell permeability, compromised barrier protection, and heightened infiltration of neutrophils and inflammatory monocytes (Polidoro et al., 2020). As a nuclear non‐histone protein bound to chromosomes, high‐mobility group box 1 protein (HMGB1) is an important mediator of inflammation. In response to pathological stimuli, HMGB1 serves as a ligand for transmembrane receptors, notably the receptor for advanced glycation end products (RAGE) and Toll‐like receptors, which in turn activate distinct intracellular signaling cascades (Wang et al., 2021). Zhan et al. demonstrated that the generation of neutrophil extracellular traps mediated by HMGB1 aggravates acute lung injury induced by intestinal I/R (Zhan et al., 2022). Deng et al. found that HMGB1 activates macrophages to secrete proinflammatory cytokines, thereby inducing acute lung injury through a Toll‐like receptor 4 (TLR4)‐dependent mechanism (Deng et al., 2013). Therefore, HMGB1 is closely associated with lung injury‐related inflammation, and the mechanisms underlying HMGB1 and MI/R‐induced lung injury remain unclear.

Electroacupuncture (EA), a traditional therapy associated with few side effects, is increasingly used as an adjunctive intervention in the ERAS protocol (Li et al., 2025; Tong et al., 2025). This integration is driven by its potential for organ protection and the enhancement of recovery processes. Numerous studies have indicated that EA can mitigate the lung injury caused by various factors (Lv et al., 2022; Zhou et al., 2023; Zou et al., 2021). Rana Dhar et al. studied the effect of EA on inflammasomes in cardiopulmonary bypass lung injury. The results demonstrated that EA inhibits inflammasome activation and caspase8‐dependent apoptosis by interfering with the reactive oxygen species (ROS)/ nuclear factor‐erythroid 2 related factor 2 (Nrf2) pathway (Dhar et al., 2019). Lou et al. found that EA alleviates lung inflammation triggered by limb I/R through suppression of the TLR4/NF‐κB signaling (Lou et al., 2020). Whether this therapeutic benefit extends to MI/R‐induced lung injury remains unknown.

This study aimed to establish a stable rat model of MI/R‐induced lung injury, to investigate whether EA preconditioning can reduce lung injury, and whether it plays a major role through the HMGB1/RAGE pathway.

## 2 MATERIALS AND METHODS

### 2.1 Animals

One hundred male SD rats (RRID: RGD_401976475) weighing 280 ± 20 g were provided by the Beijing Weitong Lihua Laboratory Animal Technology Co., Ltd. [SCXK (Beijing) 2021–0006]. The animals were housed individually in the Laboratory Animal Center of Shanghai University of Traditional Chinese Medicine, and the study protocol was approved by the institution's Animal Ethics Committee in accordance with relevant standards for experimental animal welfare (PZSHUTCM220307008). Rats were stratified by body weight and litter, then randomly assigned to groups within each stratum. All rats were kept under controlled conditions with a 12 h light/dark cycle, a temperature of 22°C–28°C, 60%–70% humidity, and adaptive feeding (SLAC Laboratory Animal Co, Shanghai, China) for 1 week (Lai et al., 2017). Animals were euthanized by carbon dioxide inhalation following the American Veterinary Medical Association guidelines for the euthanasia of animals.

Based on preliminary experiments, the expected mean difference in lung wet/dry (W/D) weight ratio between the intervention and control groups was 1.6, with a standard deviation of 0.8. Using a two‐tailed *t*‐test (*α* = 0.05, power = 90%), the required sample size was 5 rats per group. All histological and immunohistochemical evaluations were performed by investigators blinded to group allocation.

### 2.2 Experimental design

Experiment I was conducted to determine the appropriate ischemia time for establishing an MI/R model. Rats were initially allocated to two main groups according to a random number table: Sham group and I/R model group (*n* = 15 per group). Each main group was further randomly divided into three subgroups based on different ischemia durations: 30 min, 45 min, and 1 h (*n* = 5 per subgroup).

Experiment II examined the temporal effects of EA pretreatment on lung injury. Rats were randomized into the following groups: Sham group, I/R group, 30 min EA + I/R group (receiving 30‐min EA pretreatment), and 3 d EA + I/R group (receiving 3‐day EA pretreatment) (*n* = 5 per group).

Experiment III aimed to determine the changes in inflammatory factors and the main infiltrated inflammatory cells. Rats were randomized into the following groups: Sham group, I/R group, and 3 d EA + I/R group (*n* = 5 per group).

Experiment IV was designed to examine RAGE and HMGB1 expression levels in the lung tissues following EA pretreatment across different groups. Rats were randomly assigned to Sham, I/R, and 3 d EA + I/R groups (*n* = 5 per group).

Experiment V was performed to verify whether the protective effect of EA was mediated through the HMGB1/RAGE axis. Rats were randomly allocated into four groups: I/R + Vehicle, I/R + rHMGB1, EA + I/R + Vehicle, and EA + I/R + rHMGB1 (*n* = 5 per group).

### 2.3 Establishment of I/R model

The I/R model was established by transient occlusion of the left anterior descending coronary artery (LAD). In brief, the animals were anesthetized. A left thoracotomy was performed to expose the heart. The LAD was ligated 2–3 mm below the left auricle by using a 6–0 prolene suture (Lai et al., 2017). Myocardial ischemia was induced by tightening the snare and was confirmed by immediate visual observation of regional cyanosis and pallor in the anterior left ventricular wall, accompanied by ST‐segment elevation on an electrocardiogram (ECG). The ligature was maintained for the designated ischemic period (30 min, 45 min, or 1 h). Reperfusion was initiated by releasing the snare and verified by the return of a reddish hue to the previously ischemic zone and corresponding ECG changes.

The sham group experienced the same procedure, but the artery was neither occluded nor subjected to reperfusion.

### 2.4 EA pretreatment

The Zusanli (ST36) acupoint is located between the tibia and fibula, on the distal lateral side of the anterior tibial tubercle, approximately 5 mm from the tubercle. The Feishu (BL13) acupoint lies approximately 1.5 cm lateral to the posterior midline, at the level between the spinous processes of the third and fourth thoracic vertebrae. Rats receiving EA pretreatment were anesthetized with 2% isoflurane. Anesthesia was maintained at 1.5%, while bilateral ST36 and BL13 acupoints were stimulated using an electronic acupuncture device (SDZ‐IIB; Suzhou Medical Instrument Co., LTD., Suzhou, China) at 2/20 Hz for 30 min over three consecutive days, with the intensity set to induce moderate muscle contraction in the hind limb (Liu et al., 2024). The sham and I/R groups were anesthetized identically but did not receive EA stimulation.

### 2.5 Intubation‐mediated intratracheal (IMIT) administration

For intratracheal administration, anesthesia was induced with 4% isoflurane 10 min before reperfusion. After loss of the righting reflex, endotracheal intubation was performed, and anesthesia was maintained with 2% isoflurane. Either recombinant high‐mobility group box 1 protein (rHMGB1, 0.5 μg per rat) or an equal volume of normal saline (Vehicle group) was then injected intratracheally through the endotracheal tube using a 50‐μL microsyringe (Lawrenz et al., 2014).

### 2.6 Myocardial infarct size determination

After the rat heart was excised, it was rinsed with normal saline to remove blood. The surface moisture was blotted dry using clean absorbent paper. The heart was placed in a heart mold and frozen at −20°C for 15–20 min. After removal from the refrigerator, the heart was sliced transversely from the ligature into approximately 2‐mm‐thick sections using a surgical blade. The slices were immediately immersed in 1% triphenyltetrazolium chloride (TTC) solution and incubated at 37°C in the dark for 10–15 min, with gentle turning to ensure complete submersion. After staining, the slices were washed with normal saline, placed on a glass slide, and photographed. Viable myocardium stained red, whereas the infarcted area appeared pale. Subsequently, the sections were fixed in 4% paraformaldehyde (Wuhan Service Biotechnology Co., Ltd., G1101‐500ML). For each heart, five sections were examined. An investigator blinded to group allocation quantified the infarct size and left ventricular (LV) size (Wu et al., 2020).

### 2.7 Arterial blood gas analysis

Following anesthesia, a laparotomy was performed to expose the abdominal aorta. Approximately 2 mL of abdominal aortic blood was collected for blood gas analysis, and arterial partial pressure of oxygen (PaO_2_) and carbon dioxide (PaCO_2_) were measured.

Oxygenation index (OI) = PaO_2_/fraction of inspired oxygen (FiO_2_);

Barometric pressure (PB) was 760 mmHg. The water vapor pressure (PH_2_O) was 47 mmHg. The respiratory quotient (RQ) is 0.8. Alveolar‐arterial oxygen gradient (A‐aDO_2_) = (PB‐PH_2_O) × FiO_2_‐PaCO_2_/RQ‐PaO_2_.

### 2.8 Measurement of lung W/D ratio

The right middle lobe of the lungs of rats was taken for lung wet weight measurement, and then the lung tissue was dried in an incubator at 70°C for 2 days and weighed again, that is, dry weight, and the W/D ratio was calculated.

### 2.9 Lung histology analysis

The lung of rats was collected, rinsed with saline to remove blood, expanded on gauze, trimmed, and placed in paraformaldehyde tissue fixing solution (Wuhan Service Biotechnology Co., LTD, G1101‐500ML, 4:100) for 2 days. The fixed tissues were then embedded in paraffin, sectioned, and subjected to hematoxylin and eosin (H&E) staining and apoptosis immunohistochemistry.

Sections stained with H&E were scanned to calculate the lung injury score. The lung injury score was derived from the literature. For each lung, five sections were examined, and ten random fields per section were averaged to obtain the results. The specific score was divided into four criteria based on alveolar fibrin/edema, alveolar hemorrhage, septal thickening, and cellular infiltration: 0 (no damage), 1 (less than 25%), 2 (25%–50%), or 3 (>50%) (Dos Santos et al., 2022). Four criteria were combined to generate the lung injury score.

### 2.10 Immunohistochemistry

Following deparaffinization with xylene and rehydration in graded ethanol, the slides were subjected to antigen retrieval in heated citrate buffer (Wuhan Service Biotechnology Co., LTD, G1202, pH 6). The slides were rinsed after blocking, and then incubated with primary antibodies against F4/80 (Wuhan Service Biotechnology Co., LTD, GB11027, 1:1000), Cleaved Caspase‐3 (Cell Signaling Technology, #9661, 1:200), myeloperoxidase (MPO) (Wuhan Service Biotechnology Co., LTD, GB11224, 1:500) overnight at 4°C. To block endogenous peroxidase, slides were incubated with 3% hydrogen peroxide before incubation with a secondary antibody (Service Bio Cat# GB23303, RRID: AB_2811189). Slides were incubated with DAB substrate (Beyotime, Shanghai, China, #P0203) for 15 min and counterstained with hematoxylin. To quantify immunoreactivity, three to five random fields per slide were captured and analyzed using Image‐Pro Plus 6.0 (Huang et al., 2022). The result is represented by the integral optical density (IOD) SUM/area SUM.

### 2.11 Real‐time PCR analysis

Lung and heart tissues were used for total RNA extraction with a commercial kit (Sangon Biotech Co., Ltd., Shanghai, China, #B611607). 1 μg RNA was reverse transcribed into cDNA using the PrimeScript™ RT reagent Kit (TaKaRa, Dalian, China, #RR037A). Quantitative real‐time PCR was performed under the following conditions: initial denaturation at 95°C for 30 s, followed by 40 cycles of 95°C for 5 s and 60°C for 34 s. A dissociation curve analysis (95°C for 15 s, 60°C for 1 min, and 95°C for 15 s) was included to verify amplification specificity. β‐actin was used as the endogenous control. All primer sequences are provided in Table 1.

**TABLE 1:** Primer sequences.

IL‐1β	Forward	AATCTCACAGCAGCATCTCGACAAG
	Reversed	TCCACGGGCAAGACATAGGTAGC
IL‐6	Forward	ACTTCCAGCCAGTTGCCTTCTTG
	Reversed	TGGTCTGTTGTGGGTGGTATCCTC
CXCL1	Forward	GCAGACAGTGGCAGGGATTCAC
	Reversed	TGAGTGTGGCTATGACTTCGGTTTG
CXCL2	Forward	ATGCTGTACTGGTCCTGCTCCTC
	Reversed	GTCACCGTCAAGCTCTGGATGTTC
HMGB1	Forward	AGGCTGACAAGGCTCGTTATGAAAG
	Reversed	GGGCGGTACTCAGAACAGAACAAG
RAGE	Forward	CTGCCTCTGAACTCACAGCCAATG
	Reversed	TCCTGGTCTCCTCCTTCACAACTG

### 2.12 Western blot analysis

Lung tissue proteins were resolved by gel electrophoresis, transferred to membranes, and probed with primary antibodies. Following incubation with HRP‐conjugated secondary antibodies, immunoreactivity was detected by enhanced chemiluminescence (Shanghai Tanon Technology Co., LTD, 180‐501DD). Anti‐GAPDH Antibody (Abcam Cat# ab181602, RRID: AB_2630358), Anti‐HMGB1 Rabbit Pab (Service Bio Cat# GB11103, RRID: AB_3675277), and anti‐Rage Rabbit Pab (Service Bio CAT# GB11278, RRID: AB_3750688).

Goat Anti‐Rabbit IgG‐HRP (Absin Bioscience Cat# abs20002A, RRID: AB_2716554). All experiments were performed in triplicates. ImageJ software (RRID:SCR_003070) was used to quantify the western blot bands.

### 2.13 Statistical analysis

All data were analyzed with SPSS (RRID:SCR_002865). Values are presented as mean ± SEM. Student's *t*‐test was used to compare the mean values between the two groups. Statistical significance among multiple groups was determined by one‐way ANOVA, with the Student–Newman–Keuls test subsequently applied for post‐hoc analysis. *p* < 0.05 was considered statistically significant.

## 3 RESULTS

### 3.1 One hour MI/R induced stable lung injury in rat model

**FIGURE 1:**
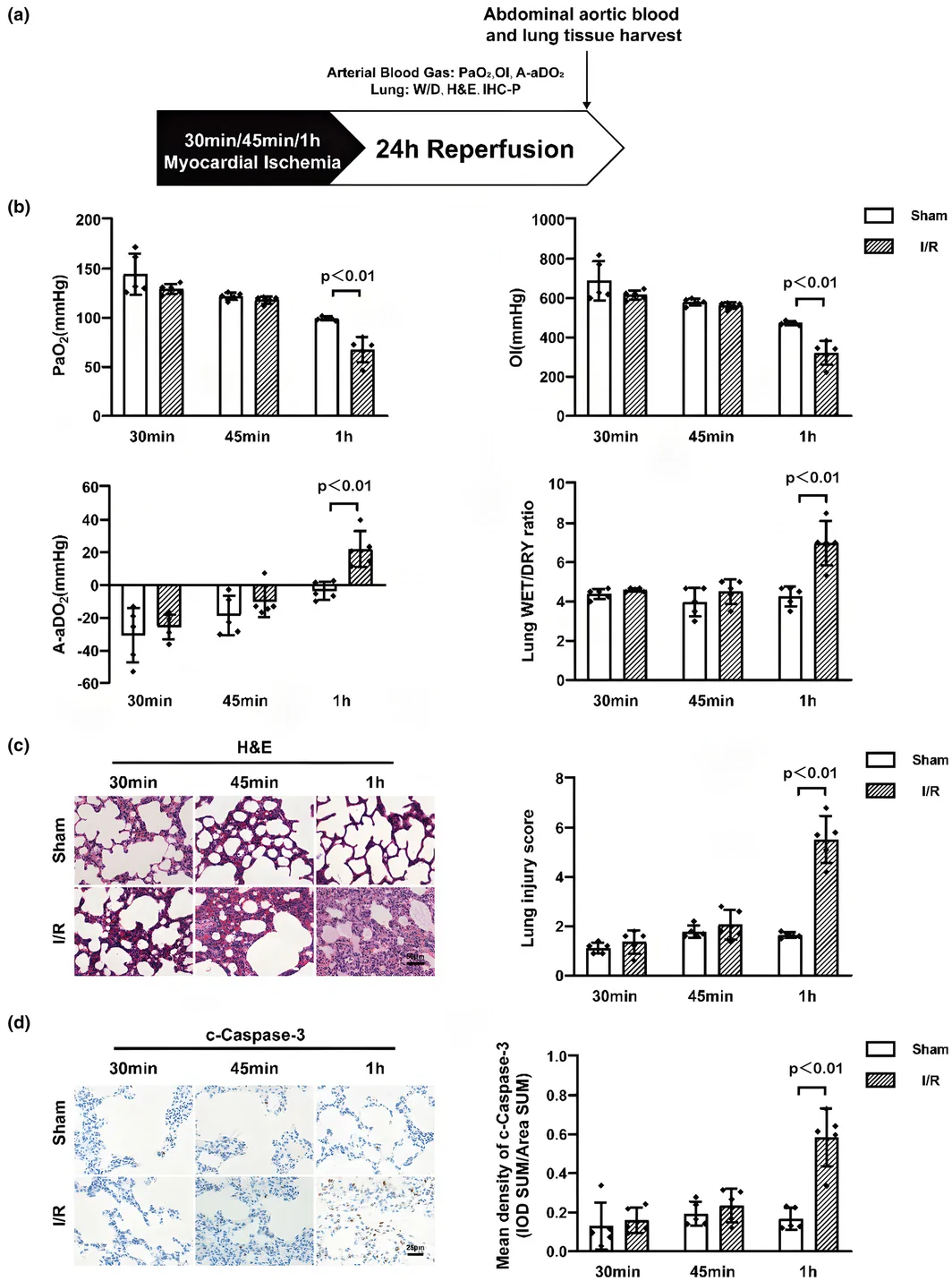
Severe lung injury occurred after 1 h of myocardial ischemia and 24 h of reperfusion in SD rats. (a) Protocol schematic for myocardial I/R and tissue harvest. (b) Arterial blood gas analysis and lung wet/dry ratio in different ischemic time groups. Statistical results of PaO2 (Upper right), OI (Upper left), A‐aDO2 (Lower right) and lung W/D ratio (Lower left) (mean ± SD, *n* = 5). (c) (Right) Representative H&E staining images of lung tissue in Sham group and I/R group with different ischemic time(scale bar,50 μm)and statistical results of (Left) lung injury score(mean ± SD, *n* = 5). (d) (Right) Representative images of c‐Caspase‐3 immunohistochemical staining of lung tissue in Sham group and I/R group with different ischemic duration (scale bar, 25 μm). Blue is the normal nucleus, brown is the positive expression of c‐Caspase‐3, and black arrow is the representative positive expression label and (Left) Statistical results of representative c‐Csapase‐3 expression (represented with integral optical density(IOD) SUM/Area SUM) (mean ± SD, *n* = 5).

Lung function, pulmonary inflammatory infiltration, and apoptosis of lung tissue in rats were measured after ischemia for 30 min, 45 min and 1 h then reperfusion for 24 h, respectively. In comparison to the Sham group, PaO2 and OI decreased significantly, but A‐aDO2 significantly increased ischemia for 1 h then reperfusion for 24 h (*p* < 0.01, *p* < 0.01, *p* < 0.01, Figure 1b); however, the difference was not statistically significant in ischemia for 30 min and 45 min (Figure 1b). These results suggest that the pulmonary function of rats decreased most significantly in myocardial ischemia for 1 h then 24 h reperfusion.

The lung W/D ratio was measured to analyze lung edema. The lung W/D ratio in ischemia for 1 h then 24 h reperfusion significantly increased compared with the sham group (*p* < 0.01). However, there were no significant differences in ischemia between 30 and 45 min (Figure 1b).

In the I/R group, lung tissue exhibited acute inflammation, including alveolar collapse, atelectasis, and varying degrees of inflammatory cell infiltration, most pronounced after 1 h of ischemia followed by 24 h of reperfusion (Figure 1c). Lung injury scores showed that lung injury increased ischemia for 1 h then 24 h of reperfusion (*p* < 0.01, Figure 1c).

Figure 1d illustrates minimal positive c‐Caspase staining (brown) in lung sections of sham animals, while a significant presence of c‐Caspase‐positive cells was observed in the rats with ischemia for 45 min and 1 h then 24 h reperfusion, indicating obvious apoptosis. Quantitative analysis demonstrated a lower count of c‐caspase‐positive cells in the 1 h ischemia/24 h reperfusion group than in the 30 min and 45 min ischemia groups (*p* < 0.01, Figure 1d).

The results demonstrated that Stable pulmonary respiratory dysfunction and severe pulmonary injury were observed in rats with myocardial ischemia for 1 h then reperfusion for 24 h.

### 3.2 Three days EA pretreatment alleviated MI/R‐induced lung injury

**FIGURE 2:**
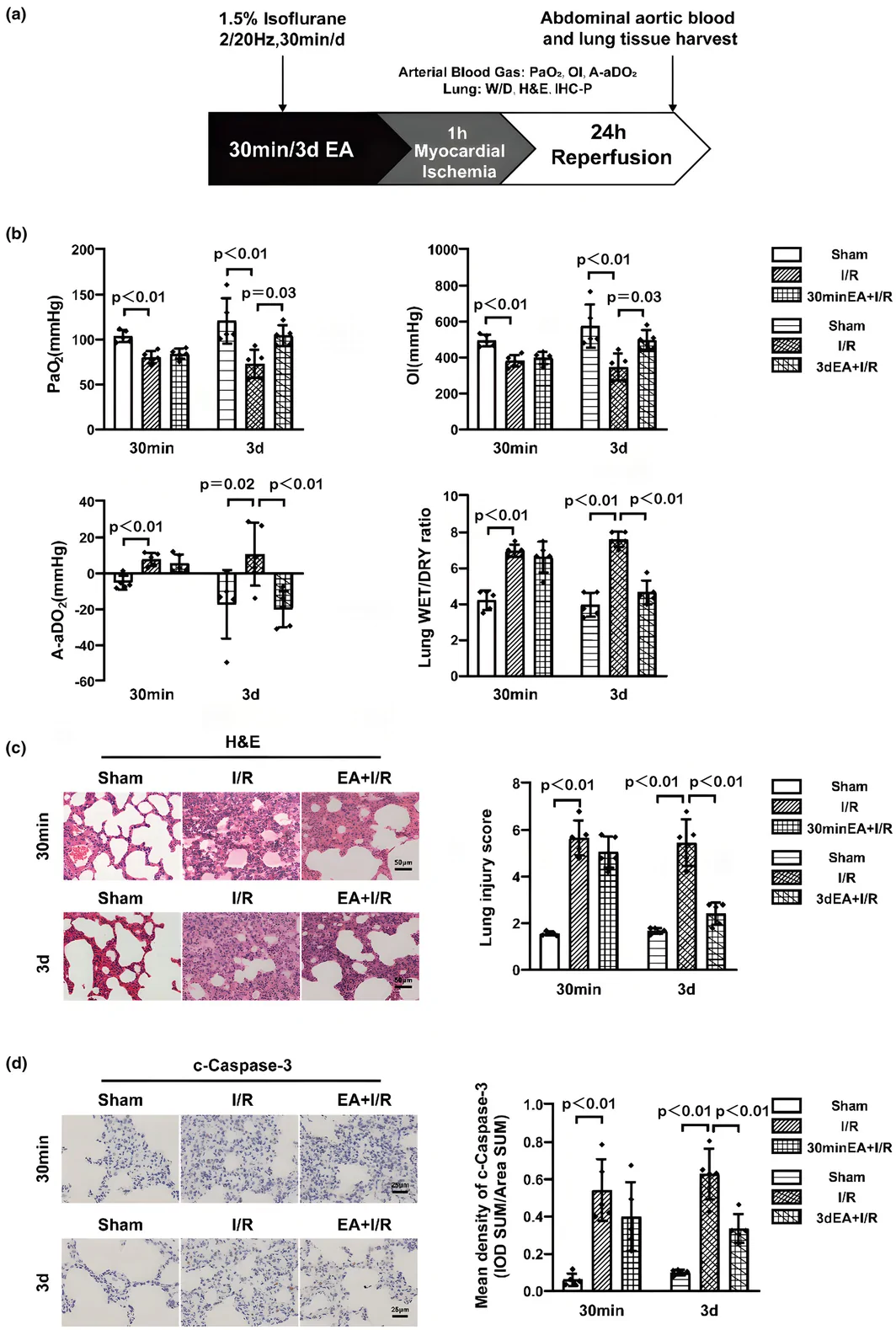
EA pretreatment for 3d can effectively improve lung injury after myocardial ischemia reperfusion in SD rats compared with pretreatment for 30 min. (a) Protocol schematic for EA pretreatment, myocardial I/R and tissue harvest. (b) Arterial blood gas analysis and lung wet/dry ratio in each group pretreated by electroacupuncture at different time. Statistical results of PaO2 (Upper right), OI (Upper left), A‐aDO2 (Lower right) and lung W/D ratio (Lower left) (mean ± SD, *n* = 5). (c) (Right) Representative staining images of lung tissue H&E in each group pretreated with electroacupuncture at different time (scale bar,50 μm) and statistical results of (Left) lung injury score (mean ± SD, *n* = 5). (d) (Right) Representative images of c‐Caspase‐3 immunohistochemical staining of lung tissues in different electroacupuncture pretreatment groups (scale bar, 25 μm). Blue is the normal nucleus, brown is the positive expression of c‐Caspase‐3, and black arrow is the representative positive expression label and (Left) Statistical results of representative c‐Csapase‐3 expression (represented with integral optical density (IOD) SUM/Area SUM) (mean ± SD, *n* = 5).

At 30 min or 3 d of EA pretreatment, compared with the sham group, the PaO2 and OI of the I/R group decreased (*p* < 0.01, *p* < 0.01; Figure 2b), but A‐aDO2 increased significantly (*p* < 0.01, *p* < 0.05; Figure 2b). The indices of blood gas analysis were not significantly different between the I/R group and 30 min EA group. However, the PaO2 and OI were increased (*p* < 0.05, *p* < 0.05, Figure 2b), and A‐aDO2 was significantly decreased in the 3 d EA group (*p* < 0.01, Figure 2b).

Compared with the sham group, the lung W/D ratio of rats in the I/R group increased significantly (*p* < 0.01, *p* < 0.01, Figure 2b). In contrast to the 3 d EA group which exhibited a significant decrease (*p* < 0.01, Figure 2b), the 30 min EA group did not differ significantly from the I/R group.

H&E staining showed that, compared with the sham group, the lung tissues of rats in the I/R group exhibited marked inflammatory cell infiltration and collapse of the alveolar architecture, resulting in a significantly higher lung injury score. (*p* < 0.01, *p* < 0.01, Figure 2c). Compared with the I/R group, there was no significant difference in lung injury scores in the 30 min EA group; however, the lung injury score of rats decreased significantly in the 3 d EA group (*p* < 0.01, Figure 2c).

C‐Caspase‐3 positive cells (brown) were scarcely observed in the sham group, while they were abundant in the I/R and EA groups (Figure 2d). The 3 d EA group exhibited a small amount of c‐Caspase‐3 staining. Quantitative analysis indicated that EA pretreatment for 3 d significantly decreased c‐Caspase‐3 positive cell counts, compared with the I/R group (*p* < 0.01, Figure 2d). The I/R and 30 min EA groups showed no significant differences. These results suggested that EA pretreatment of bilateral BL13 and ST36 for 3 days significantly reduced lung injury after myocardial I/R injury.

### 3.3 EA pretreatment inhibited inflammation in lung tissue after MI/R

**FIGURE 3:**
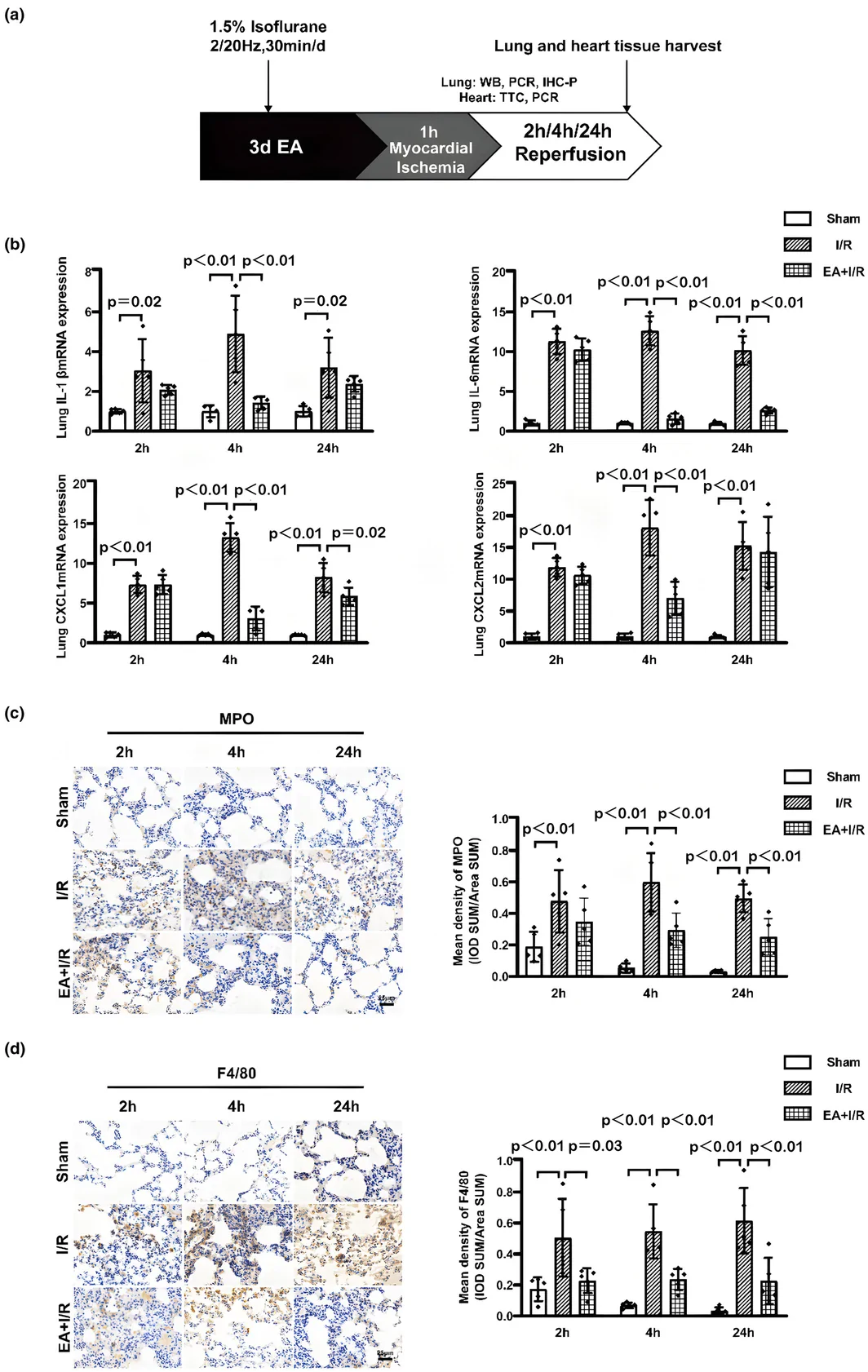
The changes of inflammatory factors and main infiltrated inflammatory cells in each group were determined by EA pretreatment for 3d and reperfusion for 2 h/4 h/24 h. (a) Protocol schematic for EA pretreatment, myocardial I/R and tissue harvest. (b) The mRNA expression levels of lung inflammatory factor gene were detected by quantitative polymerase chain reaction at different reperfusion times. Statistical results of IL‐1β (Upper right), IL‐6 (Upper left), CXCL1 (Lower right), CXCL2 (Lower left) (mean ± SD, *n* = 5). (c) (Left) MPO immunohistochemical staining images of lung tissues in different reperfusion time groups (scale bar, 25 μm). MPO positive neutrophils are shown in brown, and the black arrows are representative of the positive expression marks and (Right) statistical results of representative MPO expression(represented with integral optical density (IOD) SUM/Area SUM)(mean ± SD, *n* = 5). (d) (Left) Representative immunohistochemical staining images of lung F4/80 in different reperfusion time groups (scale bar, 25 μm). Brown is F4/80 positive macrophages, and black arrows are representative positive expression marks and (Right) statistical results of representative MPO expression(represented with integral optical density (IOD) SUM/Area SUM) (mean ± SD, *n* = 5).

The I/R group exhibited significantly increased mRNA expression of IL‐1β, IL‐6, CXCL1, and CXCL2 in rat lung tissues at various reperfusion times compared with the sham group (*p* < 0.01 for all, Figure 3b), indicating the presence of pulmonary inflammation. Compared with the I/R group, the expression levels of IL‐1β, IL‐6, CXCL1, and CXCL2 mRNA in the lung tissue decreased significantly in the EA group after I/R 4 h (*p* < 0.01 for all, Figure 3b), and the levels of IL‐6 and CXCL1 were still significantly decreased at I/R 24 h (*p* < 0.01, *p* < 0.05, Figure 3b).

Immunohistochemical staining was used to observe the main types of inflammatory cells present during lung injury. At various time points after reperfusion, the I/R group showed a significant increase in MPO and F4/80 positive cells in lung tissue compared with the sham group. (*p* < 0.01, Figure 3c *p* < 0.01, Figure 3d). Compared with the I/R group, MPO and F4/80 positive cells in the EA group decreased significantly at I/R 4 h (*p* < 0.01, Figure 3c, *p* < 0.01, Figure 3d). At I/R 24 h, there were still fewer MPO positive and F4/80 positive cells in the EA group (*p* < 0.01, Figure 3c, *p* < 0.01, Figure 3d).

Immunohistochemical staining showed that the level of HMGB1 in the lung tissue of the I/R group was significantly higher than that in the sham group (*p* < 0.01, Figure 3e). Compared with the I/R group, HMGB1 positive cells in the EA group decreased significantly at I/R 4 h (*p* < 0.01, Figure 3e).

These results suggest that pretreatment of bilateral BL13 and ST36 for 3 days can significantly attenuate the pulmonary inflammation.

### 3.4 EA pretreatment downregulated lung HMGB1/RAGE expression in MI/R model

**FIGURE 4:**
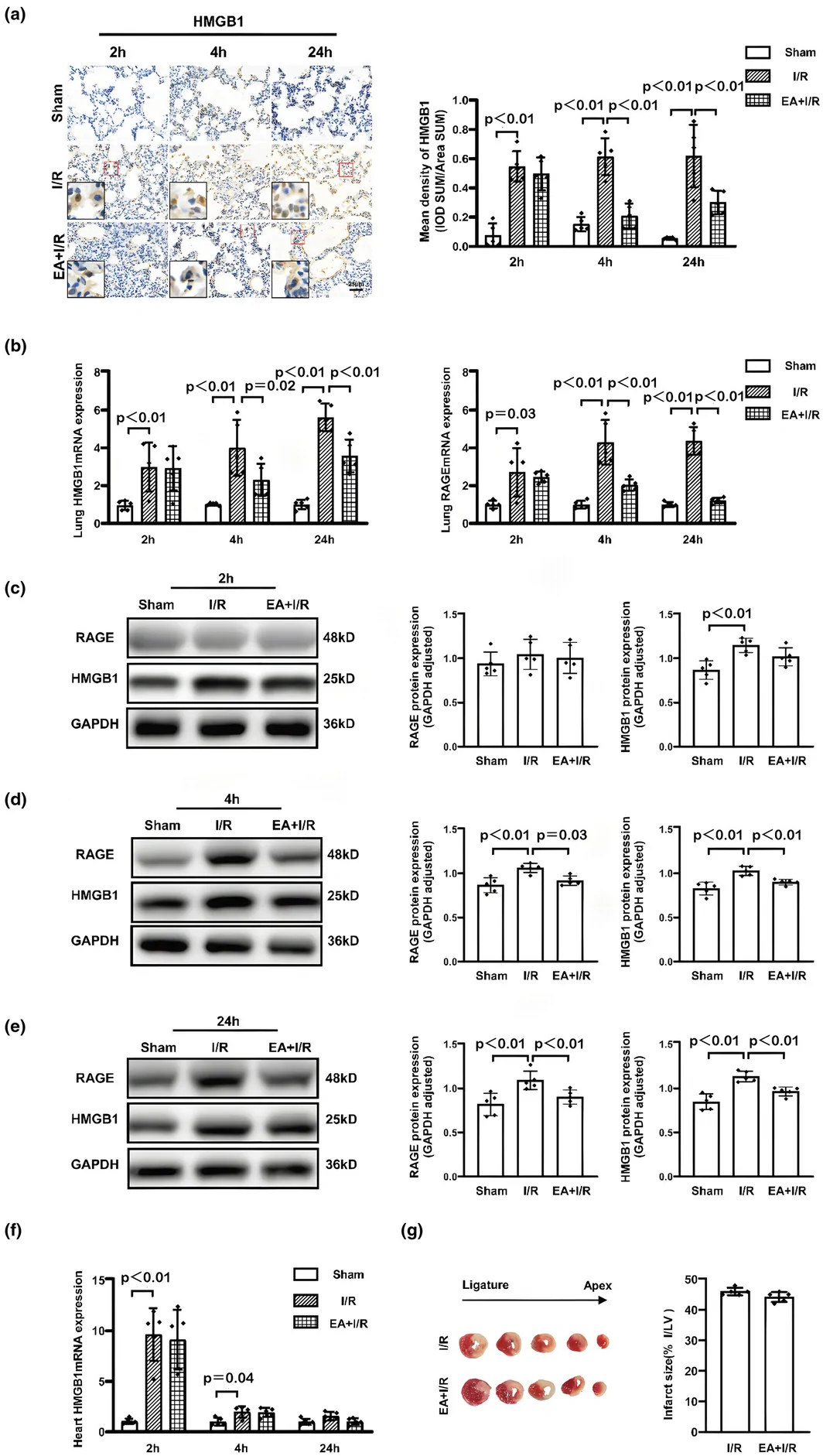
The expression levels of RAGE and HMGB1 in the lungs of each group were determined by EA pretreatment for 3d and reperfusion for 2 h/4 h/24 h. (a) (Left) Representative immunohistochemical staining images of lung HMGB1 in different reperfusion time groups (scale bar, 25 μm). Brown is HMGB1 positive macrophages, and black arrows are representative positive expression marks (the brown cells in the area marked by the red border are pulmonary macrophages) and (Right) statistical results of representative HMGB1 expression (represented with integral optical density (IOD) SUM/Area SUM)(mean ± SD, *n* = 5). (b) The mRNA expression levels of lung RAGE and HMGB1 was detected by quantitative polymerase chain reaction at different reperfusion times. Statistical results of RAGE (Right) and HMGB1 (Left) (mean ± SD, *n* = 5). (c), (d) and (e) the levels of RAGE and HMGB1 protein in lung tissues of different reperfusion time groups were detected by western blot. (Right) Representative protein band diagram. Statistical results of RAGE (Middle) and HMGB1 (Left) protein relative expression (mean ± SD, *n* = 5). (f) Statistical results of HMGB1 gene expression in heart tissues at different reperfusion times (mean ± SD, *n* = 5). (g) (Right) Representative TTC staining images of the hearts of the I/R group and the EA + I/R group after 24 h reperfusion (Red is normal heart tissue, gray is infarcted heart tissue) and (Left) statistical results (represented with infarct area (I)/left ventricular (LV) area) (mean ± SD, *n* = 5).

EA pretreatment significantly reduced the mRNA expression of HMGB1 and RAGE in the lung tissue (*p* < 0.01for all, Figure 4a). Western blot analysis revealed a significant upregulation of HMGB1 and RAGE protein levels in the lung tissue of rats in the I/R group compared with the sham group (*p* < 0.01, Figure 4b, *p* < 0.01, *p* < 0.01, Figure 4c, *p* < 0.01, *p* < 0.01, Figure 4d), indicating increased HMGB1/RAGE protein expression in MI/R‐induced lung injury. The results showed that the expressions of HMGB1 and RAGE in lung tissue decreased in the EA + I/R group at 4 h and 24 h compared with the I/R group (*p* < 0.05, *p* < 0.01, Figure 4c, *p* < 0.05, *p* < 0.01, Figure 4d).

To identify the source of HMGB1, PCR was used to detect the HMGB1 expression in the heart. The results showed that, compared with the sham group, the HMGB1 expression in the heart of the I/R group was upregulated at the early stage (*p* < 0.01, Figure 4e), and it was still upregulated after I/R 4 h (*p* < 0.05, Figure 4e), but no significant difference was observed after I/R 24 h, and the expression of HMGB1 in the EA group was not significantly different. At the same time, there was no significant difference in the infarct size (Figure 4f). These results indicate that the increase in HMGB1/RAGE in the lung tissue does not originate from the heart.

These results suggest that EA pretreatment downregulated the expression of HMGB1/RAGE in the lungs of the MI/R model and indicated that the elevated HMGB1/RAGE in the lungs did not originate from the heart.

### 3.5 IMIT rHMGB1 reverses the effect of EA

**FIGURE 5:**
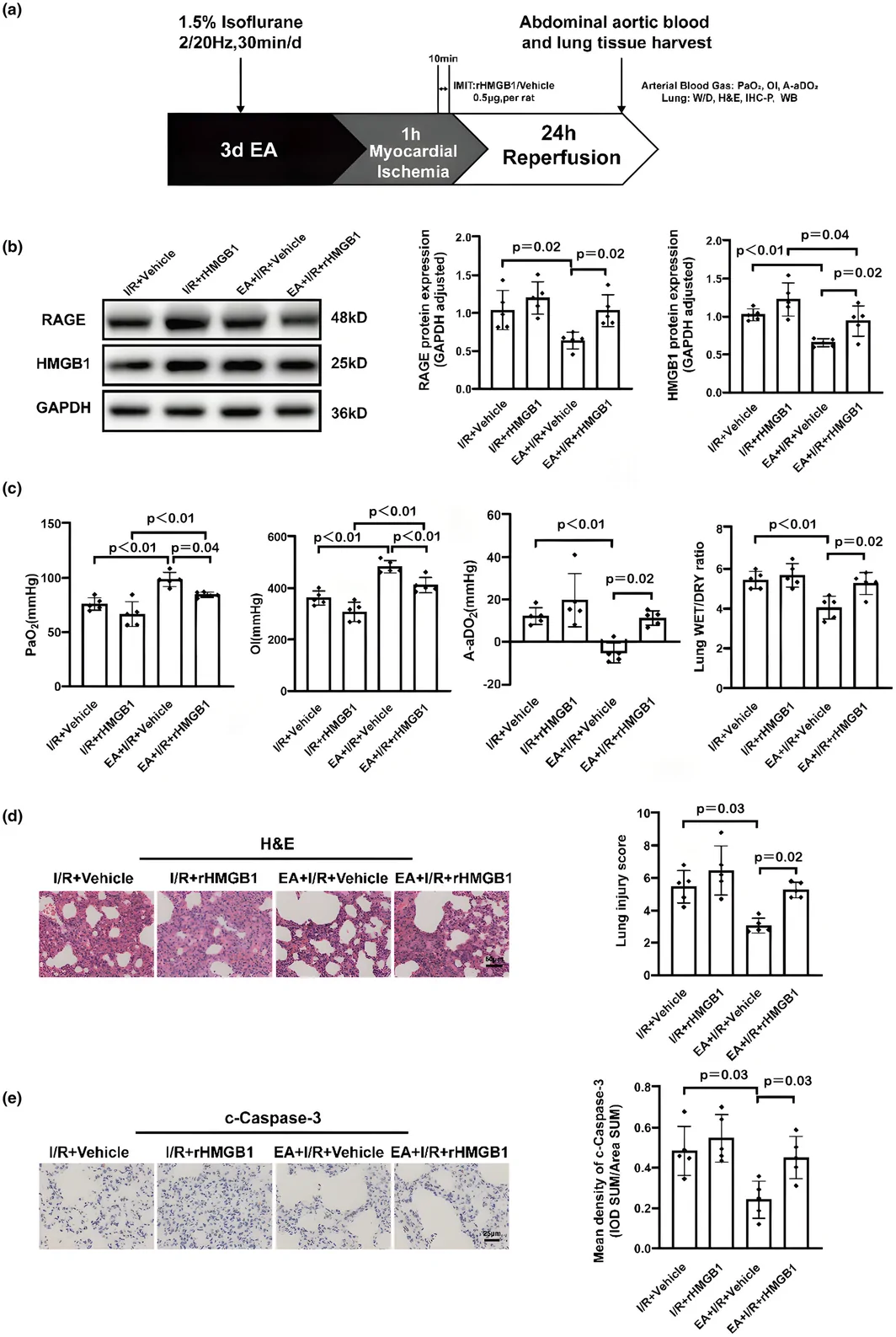
IMIT of rHMGB1 aggravated lung injury after myocardial I/R in rats to a certain extent and reversed the therapeutic effect of EA. (a) Protocol schematic for IMIT and tissue harvest. (b) The levels of RAGE and HMGB1 protein in lung tissues of each group were detected by western blot. (Right) Representative protein band diagram. Statistical results of RAGE (Middle) and HMGB1 (Left) protein relative expression (mean ± SD, *n* = 5). (c) Statistical results of arterial blood gas analysis and lung wet/dry ratio in each group (mean ± SD, *n* = 5). (d) (Right) Representative H&E staining images of lung tissues in each group (scale bar,50 μm) and (Left) statistical results of lung injury score(mean ± SD, *n* = 5). (e) (Right) Representative images of c‐Caspase‐3 immunohistochemical staining of lung tissue in each group (scale bar, 25 μm). Blue is the normal nucleus, brown is the positive expression of c‐Caspase‐3, and black arrow is the representative positive expression label and (Left) Statistical results of representative c‐Csapase‐3 expression (represented with integral optical density (IOD) SUM/Area SUM) (mean ± SD, *n* = 5).

To increase the expression of HMGB1 in the lungs and observe the effect of EA. After IMIT of rHMGB1, pulmonary function and acute inflammatory reactions in the lungs were detected. Western blot results showed that the expression of HMGB1 mRNA and RAGE mRNA in the lung tissue of the I/R + rHMGB1 group were higher than those in the I/R+ Vehicle group, but the difference was not statistically significant. However, compared with the EA + I/R + Vehicle group, the expression of HMGB1 mRNA and RAGE mRNA in the lung tissue of the EA + I/R + rHMGB1 group was significantly increased (*p* < 0.05, *p* < 0.05, Figure 5b).

To increase the expression of HMGB1 in the lungs and observe the effect of EA. After IMIT of rHMGB1, pulmonary function and acute inflammatory reactions in the lung were detected. Western blot results showed that compared with the I/R + Vehicle group, the expression of HMGB1 and RAGE in EA + Vehicle lung tissue decreased significantly (*p* < 0.01, *p* < 0.01, Figure 5b). Compared with the EA + I/R + Vehicle group, the expression of HMGB1, RAGE, PaO2, and OI in the EA + I/R + rHMGB1 group decreased significantly (*p* < 0.01 for all, Figure 5b, Figure 5c). The lung W/D of rats in the EA + I/R + rHMGB1 group increased significantly compared with that in the EA + I/R + Vehicle group (*p* < 0.05, Figure 5c). The lung H&E staining results showed that, compared with the EA + I/R + Vehicle group, the lung injury of rats in the EA + I/R + rHMGB1 group was significantly increased (*p* < 0.05, Figure 5d). Immunohistochemical staining of the lung tissue showed that the number of positive cells in the EA + I/R + rHMGB1 group increased significantly (*p* < 0.05, Figure 5e).

## 4 DISCUSSION

Our main results revealed that SD rats exhibited obvious lung injury following MI/R after 1 h of myocardial ischemia and reperfusion for 24 h. EA preconditioning for 3 days significantly improved lung injury after MI/R and the pulmonary inflammatory response. HMGB1 plays a key role in reducing EA‐induced lung injury after MI/R.

The first and most important objective was to establish a stable animal model. By reviewing the relevant basic research literature, the results showed that there was no uniform standard for an animal model of MI/R‐induced lung injury in rats. Meanwhile, clinical studies have shown that the incidence of MPCs in cardiac surgery increases with time, and lung injury‐related symptoms can appear on the 2nd day after surgery (Lagier et al., 2019; Touw et al., 2018). In a study, SD rats were selected for 30 min ischemia (Chen et al., 2022). Huang et al. used an SD rat model with 1 h of ischemia then reperfusion (Huang et al., 2020). Wang et al. performed reperfusion after ischemia for 1 h (Wang et al., 2018). Their results showed that various durations of ischemia cause lung injury to a certain extent. Therefore, in this study, male SD rats were subjected to myocardial ischemia for 30 min, 45 min, and 1 h, followed by 24 h of reperfusion, to simulate MI/R‐induced lung injury in clinical cardiac surgery. Our results demonstrated that, compared with ischemia in the 30 min and 45 min groups, pulmonary respiratory function decreased, pulmonary edema aggravated, and pulmonary pathological changes (including alveolar fibrin/edema, alveolar hemorrhage, thickening of the pulmonary septum, and cell infiltration) and apoptosis increased most significantly after 1 h of myocardial ischemia and reperfusion for 24 h. In other words, a stable rat model of lung injury resulting from MI/R was created through 1 h of myocardial ischemia and subsequent reperfusion.

Research indicates that EA is effective in mitigating lung injury from multiple causes (Lin et al., 2020; Yu et al., 2013; Zhang et al., 2022). Previous research has demonstrated that EA at the Neiguan (PC6) and Hegu (LI4) acupoints could reverse pulmonary inflammation, oxidative damage, and apoptosis induced by cardiopulmonary bypass (CPB) (Ma et al., 2017). EA pretreatment of ST36 and BL13 acupoints for 5 days suppressed NLRP3 inflammasome activation and alleviated inflammatory lung injury after CPB (Huang et al., 2019). Research indicates that EA mitigates lung injury caused by CPB through its significant anti‐apoptotic action, which involves the inhibition of the ROS/Nrf2/NLRP3 inflammasome pathway (Dhar et al., 2019). In our experiment, EA was applied to pretreat bilateral BL13 and ST36 points to observe its therapeutic effects on lung function, pulmonary edema, and lung tissue injury in rats with MI/R. Our results showed that 3 days of preoperative EA stimulation (2/20 Hz, 30 min/day, and denser waves) could significantly improve MI/R‐induced lung injury. This preconditioning strategy aligns with the ERAS principle of preoperative optimization to reduce postoperative complications and potentially facilitate faster recovery.

MI/R induces a systemic inflammatory response, with inflammatory cells infiltrating the lungs and releasing ROS and pro‐inflammatory mediators, ultimately damaging alveolar epithelial and pulmonary capillary endothelial cells. Research has shown that HMGB1 is expressed in the cytoplasm of pulmonary macrophages and neutrophils during ventilator‐induced lung injury. Blocking HMGB1 limited microvascular permeability and neutrophil flow into the alveolar lumen and reduced the level of TNF‐α (Ogawa et al., 2006). Studies have further demonstrated that HMGB1 disrupts microvascular endothelial cell barrier function during septic acute lung injury by activating the RhoA/ROCK1 pathway via RAGE (Zhao et al., 2021). Wang et al. showed that HMGB1 drives sepsis‐induced acute lung injury by regulating macrophage function, specifically through absent in melanoma 2 inflammasome activation and M1 polarization mediated by TLR2/TLR4/RAGE/NF‐κB signaling (Wang et al., 2020). Given that HMGB1 acts as a pivotal mediator of inflammatory cell recruitment in various forms of acute lung injury, it likely serves as a plausible link between the systemic inflammation triggered by MI/R and the ensuing pulmonary damage. Moreover, because EA has been reported to suppress systemic inflammatory responses (Lv et al., 2022; Zhou et al., 2023), we reasoned that EA might protect against MI/R‐induced lung injury by modulating the HMGB1/RAGE pathway. To test this, we measured the inflammatory response and the HMGB1/RAGE pathway. The results showed that the number of pulmonary macrophages and neutrophils, as well as the expression of IL‐6, CXCL1, and CXCL2, increased in the MI/R group, suggesting lung inflammation. In addition, HMGB1/RAGE expression increased significantly. Three days EA pretreatment effectively reduced lung inflammation and the expression of HMGB1/RAGE in lung tissue.

By detecting the content of HMGB1 in the heart, we observed that HMGB1 in the heart only increased at the early stage of inflammation, and there was little at 24 h, which suggested that the increased HMGB1 in the lung did not originate from the heart at reperfusion for 24 h. In addition, we pretreated rats with EA BL13 and ST36 for 3 days, which produced no change in the infarct area of the heart but reduced the HMGB1 expression in the lung tissue. Therefore, our results indicate that increased HMGB1 levels in the lungs are not derived from the heart.

Additionally, we assessed the effect of EA following intratracheal administration of rHMGB1. The results showed that intratracheal administration of rHMGB1 aggravated pulmonary respiratory dysfunction, pulmonary edema, and lung injury to a certain extent. EA significantly improved this injury and reduced the expression of HMGB1 and RAGE mRNA in the lung tissue. However, the effect of EA was attenuated following the same injection of rHMGB1. Li et al. identified the HMGB1/PI3K/Akt/mTOR pathway as a key regulator in dendritic cells during acute lung injury. This pathway was shown to participate in the pathological process by modulating dendritic cell maturation and function (Li et al., 2020). Our experimental results also indicate that inhibition of HMGB1 expression in the lung can attenuate lung inflammation and reduce injury after myocardial I/R.

Nevertheless, this study has several limitations. First, while we preliminarily validated the critical role of the HMGB1/RAGE pathway in the protective effects of EA through intratracheal administration of rHMGB1, reverse validation using gene knockout was not performed, which to some extent limits the causal certainty of our conclusions. Second, the sample size was relatively small, and all experiments were conducted in an animal model; therefore, caution is warranted when extrapolating these findings to clinical settings. Future large‐scale, multicenter clinical studies are necessary to validate the pulmonary protective effects of EA pretreatment.

## 5 CONCLUSION

In summary, myocardial ischemia for 1 h and reperfusion for 24 h established a stable rat lung injury model. EA preconditioning could inhibit the expression of HMGB1/RAGE in the lungs to exert its anti‐inflammatory and pulmonary protective effects in MI/R‐induced lung injury. This anti‐inflammatory mechanism may help reduce postoperative pulmonary complications and support enhanced recovery after surgery, underscoring the broader potential of EA in perioperative organ protection.

## AUTHOR CONTRIBUTIONS

Conceptualization, C.X., J.Z.; Methodology, C.X., Y.Y.; Visualization, J.Z.; Data curation, J.Z., Y.Z.; Formal analysis, J.Z., Y.Z.; Writing—original draft, C.X., J.Z.; Project administration, Y.Y., H.C.; Writing—review and editing, H.C., J.S.

## FUNDING INFORMATION

This work was supported by the National Natural Science Foundation of China under Grant [82074157, 82441058, 82304924, 81603702, 82204869 and 82204868].

## CONFLICT OF INTEREST STATEMENT

The authors report there are no competing interests to declare.

## ETHICS STATEMENT

The study protocol was reviewed by the institution's Animal Ethics Committee in accordance with relevant standards for experimental animal welfare (PZSHUTCM220307008).

## Data Availability

Corresponding authors may provide data and materials.
